# Changes in Tea Plant Secondary Metabolite Profiles as a Function of Leafhopper Density and Damage

**DOI:** 10.3389/fpls.2020.00636

**Published:** 2020-05-29

**Authors:** Eric R. Scott, Xin Li, Ji-Peng Wei, Nicole Kfoury, Joshua Morimoto, Ming-Ming Guo, Amma Agyei, Albert Robbat, Selena Ahmed, Sean B. Cash, Timothy S. Griffin, John R. Stepp, Wen-Yan Han, Colin M. Orians

**Affiliations:** ^1^Department of Biology, Tufts University, Medford, MA, United States; ^2^Tea Research Institute, Chinese Academy of Agricultural Sciences, Hangzhou, China; ^3^Department of Chemistry, Tufts University, Medford, MA, United States; ^4^Food and Health Lab, Department of Health and Human Development, Montana State University, Bozeman, MT, United States; ^5^Friedman School of Nutrition and Policy, Tufts University, Medford, MA, United States; ^6^Department of Anthropology, University of Florida, Gainsville, FL, United States

**Keywords:** *Camellia sinensis*, *Empoasca onukii*, secondary metabolites, herbivory, induced responses, plant VOCs, catechins, crop quality

## Abstract

Insect herbivores have dramatic effects on the chemical composition of plants. Many of these induced metabolites contribute to the quality (e.g., flavor, human health benefits) of specialty crops such as the tea plant (*Camellia sinensis*). Induced chemical changes are often studied by comparing plants damaged and undamaged by herbivores. However, when herbivory is quantitative, the relationship between herbivore pressure and induction can be linearly or non-linearly density dependent or density independent, and induction may only occur after some threshold of herbivory. The shape of this relationship can vary among metabolites within plants. The tea green leafhopper (*Empoasca onukii*) can be a widespread pest on tea, but some tea farmers take advantage of leafhopper-induced metabolites in order to produce high-quality “bug-bitten” teas such as Eastern Beauty oolong. To understand the effects of increasing leafhopper density on tea metabolites important for quality, we conducted a manipulative experiment exposing tea plants to feeding by a range of *E. onukii* densities. After *E. onukii* feeding, we measured volatile and non-volatile metabolites, and quantified percent damaged leaf area from scanned leaf images. *E. onukii* density had a highly significant effect on volatile production, while the effect of leaf damage was only marginally significant. The volatiles most responsive to leafhopper density were mainly terpenes that increased in concentration monotonically with density, while the volatiles most responsive to leaf damage were primarily fatty acid derivatives and volatile phenylpropanoids/benzenoids. In contrast, damage (percent leaf area damaged), but not leafhopper density, significantly reduced total polyphenols, epigallocatechin gallate (EGCG), and theobromine concentrations in a dose-dependent manner. The shape of induced responses varied among metabolites with some changing linearly with herbivore pressure and some responding only after a threshold in herbivore pressure with a threshold around 0.6 insects/leaf being common. This study illustrates the importance of measuring a diversity of metabolites over a range of herbivory to fully understand the effects of herbivores on induced metabolites. Our study also shows that any increases in leafhopper density associated with climate warming, could have dramatic effects on secondary metabolites and tea quality.

## Introduction

Insect herbivores have the potential to induce chemical changes in the plants they feed on, with notable implications for ecological interactions and crop quality in agricultural systems. Induced plant responses to herbivores include increased production of secondary metabolites that reduce herbivore feeding or fitness (Karban et al., 1997), or upregulation of indirect defenses such as volatile organic compounds or extrafloral nectar that attract natural enemies (Arimura et al., 2005). As global environmental change impacts herbivore populations (Berggren et al., 2009; Bebber et al., 2013), it is also likely to impact induced plant responses including the production of secondary metabolites (DeLucia et al., 2012).

Most studies elucidating induced plant responses to herbivores have drawn conclusions from comparisons of plants with no damage to plants with damage at a single level. However, plants growing in the wild or agricultural settings are exposed to a range of herbivory. Several studies have found variation in the relationship between the extent of herbivore damage and plant responses induced by this damage. For example, Underwood (2000) found a threshold in the amount of damage required to induce resistance in soybean. This threshold varied among soybean genotypes with cultivars responding at lower or higher levels of herbivory and one cultivar that did not show induced resistance even at 90% leaf damage.

Furthermore, the relationship between herbivore pressure and secondary metabolite concentration often varies among individual compounds resulting in a change in metabolite profiles with increasing herbivory (Horiuchi et al., 2003; Shiojiri et al., 2010; Cai et al., 2012, 2014). For example, Shiojiri et al. (2010) demonstrated that volatile compounds induced by diamondback moth (*Plutella xylostella*) larvae feeding on cabbage varied in direction and magnitude of induction. Specifically, the metabolite sabinene was produced at a higher concentration with 5% damage compared to 15 and 30% damage while emission of (*Z*)-3-hexenyl acetate was induced only at 30% damage. Similarly, Horiuchi et al. (2003) identified only 5 out of 10 compounds induced by *Tetranychus urticae* as being induced in a density-dependent manner (increasing concentration with increasing herbivore density). By investigating the impact of a range of herbivore densities on multiple classes of metabolites, we can develop a comprehensive understanding of the impact of herbivores on plant chemistry.

Herbivore density-dependent changes in plant chemistry have an important role in ecological interactions. For example, herbivores may respond to induced volatile profiles as ecological cues for conspecific density, being attracted to a volatile profile emitted by plants with low or moderate infestations, but repelled by a volatile profile produced by heavily infested plants (Horiuchi et al., 2003; Robert et al., 2012). Plant volatile profiles also serve as a signal of host density for parasitoids (Geervliet et al., 1998; Girling et al., 2011) and even hyperparasitoids (Cusumano et al., 2019). In agricultural systems, the quality of many crops is highly dependent on secondary metabolite profiles that impact flavor and nutritional quality for humans (Ahmed et al., 2014; Scott and Orians, 2018; Wüst, 2018; Johnson et al., 2019). Reductions in crop quality have important economic consequences for farmers that can outweigh reductions in yield (Kawasaki and Uchida, 2016). In integrated pest management, an economic threshold is the density of a pest at which some treatment will result in an economic return mostly based on crop yield (Higley and Pedigo, 1996). Understanding the relationship between herbivory, secondary metabolite induction, and crop quality would lead to more comprehensive economic thresholds for herbivory compared to taking only yield into account.

Tea is a globally important specialty crop produced from young leaves of the tea plant, *Camellia sinensis* (L.) O. Kuntze. Tea quality is important for consumers and farmer livelihoods (Ahmed et al., 2014; Boehm et al., 2019) and is influenced not only by the total concentration of secondary metabolites in tea, but also by the relative proportion of different metabolites (metabolite profile) (Zeng et al., 2019). Tea secondary metabolites are known to vary as a function of multiple environmental and management factors (Ahmed et al., 2019) including plant genotype (Cherotich et al., 2013; Chen et al., 2018; Mu et al., 2018), shade (Sano et al., 2018), elevation (Han et al., 2017; Kfoury et al., 2018b), drought (Scott et al., 2019), precipitation (Ahmed et al., 2014; Kowalsick et al., 2014), temperature (Lee et al., 2010), model of agricultural production (Ahmed et al., 2013; Han et al., 2018), microbes (Singh et al., 2010), and numerous pest insects (Scott and Orians, 2018). These environmental factors are shifting with global change including climate change and are impacting tea quality (Ahmed et al., 2019).

The tea green leafhopper (*Empoasca onukii* Matsuda) is a major pest on tea (Mao et al., 2014). *Empoasca onukii* are cell rupture feeders and inject a watery saliva containing a suite of digestive and oxidative enzymes into plant tissues while feeding (Backus et al., 2005; Jin et al., 2012). Leafhopper feeding on tea causes a set of symptoms known as “hopperburn,” including yellowing (chlorosis), thickening of leaves, occasionally necrosis at leaf margins, and sometimes leaf abscission. In Taiwan and Southern China, *E. onukii* can reduce tea yields by 15–20% (Fu et al., 2014). Leafhopper damage also induces the production of volatiles by tea plants (Cai et al., 2014), with implications for crop quality including flavor. In fact, farmers in some regions take advantage of changes in volatiles induced by *E. onukii* to improve the aroma of processed tea by maintaining specific levels of leafhopper herbivory in their tea production systems (Kawakami et al., 1995; Cho et al., 2007; Mei et al., 2017; Scott and Orians, 2018). This style of “bug-bitten” tea originates in Taiwan with a tea known as Eastern Beauty oolong (Scott and Orians, 2018). In a growth chamber study, Cai et al. (2014) investigated the effects of low and high densities of *E. onukii* on tea volatile metabolites and found that higher densities were associated with higher volatile emission.

Leafhoppers such as *E. onukii* are expected to increase their population densities with climate change (Masters et al., 1998; Bale et al., 2002; Baker et al., 2015). *Empoasca onukii* currently has 9–15 overlapping generations per year in China and Taiwan and 5–8 generations per year in more temperate Japan (Fu et al., 2014). Although it is known that *E. onukii* induces changes in tea plant chemistry, the effects of a range of *E. onukii* densities on the induction of volatiles is unknown, and studies on leafhopper induction of both volatile and non-volatile metabolites are lacking. It is also unclear how these responses translate to studies in the field. This study seeks to address the aforementioned research gaps.

In this study, we manipulated leafhopper density on potted tea plants grown outdoors to determine the impact of leafhopper herbivory on the concentrations of volatile and non-volatile secondary metabolites that determine tea quality. In addition to the impact on tea quality, the relationship between leafhopper damage and secondary metabolite concentrations is important for ecological interactions, such as attracting natural enemies (Gao et al., 2004).

## Materials and Methods

### Leafhopper Collection

Tea plants are commonly attacked by a single species of leafhopper, *Empoasca onukii* Matsuda (Mao et al., 2014). Leafhoppers were reared from eggs collected from tea fields and supplemented with nymphs aspirated from tea leaves in the field. *Empoasca onukii* lays its eggs under the epidermis of young tea stems, and eggs were found on field plants using a portable version of the Simplified Leafhopper Egg Detection by Autofluorescence (SLEDA) method described by Herrmann and Böll (2004). Under the light of a blue LED flashlight, leafhopper eggs fluoresce green and chlorophyll fluoresces red. By wearing goggles that block blue light (Ultra-spec 2000 S0360X, Uvex, Fürth, Germany), leafhopper eggs in the tea shoots are visible as small green dots (Supplementary Figure S1). Shoots with eggs were cut and placed in hydrated floral foam inside of a mesh bug dorm (MegaView Science, Taichung, Taiwan). First instar nymphs were found on the tea shoots in the bug dorm within a week. This lab-hatched population was supplemented with leafhopper nymphs aspirated from tea plants in the field. Leafhoppers were fed by replacing the tea shoots in the floral foam regularly. Leafhopper eggs and nymphs were collected from several tea cultivars at Shanfu Tea Company in Shaxian, Fujian Province, China.

### Experimental Approach

In the summer of 2017, we assessed the effect of leafhopper density on plant chemical responses using 2-year-old potted tea plants of the Qing Xin Da Mao (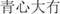) cultivar at the Shanfu Tea Company in Shaxian, Fujian Province, China. Each pot contained 3–4 plants that were propagated from cuttings and grown outdoors. Qing Xin Da Mao was chosen because it is a popular cultivar for production of Eastern Beauty oolong (Perin, 2019). On June 19, 20 pots were individually covered with mesh fabric bags (<0.25 mm mesh) after removing any visible insects. On June 25, leafhopper nymphs (2nd–4th instars) from the lab colony were introduced to the potted plants in the mesh bags based on randomly assigned density treatments. Leafhopper density treatments were chosen based on preliminary surveys of leafhopper density in tea fields (unpublished). Leafhopper density on Qing Xin Da Mao plants in a field at Shanfu tea company was 0.24 ± 0.12 leafhoppers per young leaf. The leafhopper density in the fields was considered low according to the farm manager, so our treatments were 0, 0.5, 1, 1.5, or 2.0 insects per young leaf, which bracketed the field density. The potted tea plants had an average of 12.7 young leaves and the number of leafhoppers added ranged from 0 to 30. Because leafhoppers can die during transfer (personal observation), and leafhopper nymphs may have hatched from eggs already present on the plants, we used the final density of leafhoppers at the end of the experiment for all analyses. We terminated the experiment after 4 days (June 29), when plants in high density treatments showed symptoms of hopperburn. Four days is well known to be sufficient to observe induced chemical responses in tea plants (Cai et al., 2014; Li et al., 2018; Liao et al., 2019). At the end of the experiment (June 29) we sampled volatiles and collected leaves for non-volatile analysis.

Volatiles were sampled for 2 h from a representative leaf in each pot by direct contact sorptive extraction (DCSE) (Kfoury et al., 2017). We performed DCSE by placing a polydimethylsiloxane (PDMS) coated magnetic stir bar (Twister^®^ brand, Gerstel, Mülheim an der Ruhr, Germany) on the abaxial surface of a leaf and holding it in place with two small (∼2.5 mm dia.) neodymium magnets on the adaxial surface of the leaf. During DCSE sampling, volatile compounds from the headspace and leaf surface are absorbed by the PDMS (Kfoury et al., 2017). This method was preferred over headspace methods because it allowed us to easily sample all plants in the study simultaneously with minimal equipment in the field. A field blank was collected by attaching a PDMS stir bar to a metal binder clip attached to a wooden stake at a height and distance from other plants similar to the PDMS stir bars on leaves. After 2 h, we removed the PDMS stir bars from leaves, sealed them in vials and shipped them to Tufts University for analysis. After sampling volatiles, the leaf sampled by DCSE was marked using a permanent marker on the petiole, and we harvested all young leaves and scanned the abaxial surfaces on a flatbed scanner (Perfection V19, EPSON, Suwa, Japan). Because no drying oven was available at this field site, we microwaved the leaves on medium power for 2 min to stop enzymatic activity, and then continued to microwave 1 min at a time, cooling the samples to room temperature between bouts, until dry (Ahmed, 2011).

### Image Analysis of Visible Leaf Damage

In addition to measuring the final density of leafhoppers at the end of the experiment we quantified visible leaf damage (stippling and browned tissue) as determined through pixel classification by machine learning. Using the scanned images of leaves, we created image files of individual leaves with petioles and any leaf folds or dark shadows manually edited out. Estimation of percent damaged leaf area was accomplished with the Trainable Weka Segmentation (TWS) plugin in FIJI (Schindelin et al., 2012; Schneider et al., 2012; Arganda-carreras et al., 2017). The TWS plugin is a machine learning tool that performs supervised pixel classification. To train the classifier, a random subset of 30 leaves was chosen and regions of interest were selected on each leaf representing damaged leaf area, undamaged leaf area, and background. Training features included the original image (hue, saturation, and brightness) as well as the Hessian, Sobel, variance, minimum, median, anisotropic, and bilateral filters provided in the plugin settings. The minimum and maximum sigmas were 2 and 16, respectively (Arganda-carreras et al., 2017). The training process was iterative and once we were satisfied with its performance on the training set, it was applied to a test set of 10 images to evaluate classifier performance. This test produced a Kappa coefficient of 0.998, indicating high accuracy. The final classifier was applied to all leaf images and numerical results were extracted using a custom script. Percent damage was calculated as damaged/(damaged + undamaged). Mean percent damaged leaf area was calculated for each pot. Additionally, we recorded damage to the focal leaf sampled by DCSE for each pot.

One low density leafhopper pot exhibited unusually high leaf necrosis (13.7% mean leaf damage at 0.33 leafhoppers/young leaf). Visual inspection revealed that two leaves were nearly entirely necrotic — damage that was unlikely to be due to direct effects of leafhopper feeding. This pot was therefore excluded from the experiment. As a consequence, a total of 19 pots with varying densities and levels of damage were used for further analyses.

### Plant Secondary Chemistry

#### Volatiles by Gas Chromatography-Mass Spectrometry

Analysis of plant volatiles was done using established techniques (Kfoury et al., 2017). Analyses were performed on an Agilent 6890/5975 GC-MS (Santa Clara, CA) fitted with a MultiPurpose autosampler (Gerstel) and a 30 m × 250 μm × 0.25 μm RXI-5MS column (Restek). Prior to analysis by gas chromatography-mass spectrometry (GC-MS), PDMS stir bars used in DCSE sampling were directly spiked with 1 μl of 10 μg/ml naphthalene-d_8_ (Restek, Bellefonte, PA, United States) as an internal standard. A thermal desorption unit (TDU, Gerstel GmbH, Müllheim an der Ruhr, Germany) provided spitless transfer of volatiles from the PDMS stir bars into a programmable temperature vaporization inlet (CIS, Gerstel) held at −100°C. Under helium gas flow (50 ml/min), the TDU was held at 40°C for 0.7 min, then heated to 275°C at 600°C/min and held at 275°C for 3 min. After 0.1 min the CIS was heated to 275°C at 12°C/min and held for 5 min. The GC column was heated at 40°C for 1 min, then heated to 280°C at a rate of 5°C/min with constant flow of helium at 1.2 ml/min. The ion source and quadrupole temperatures were set at 230 and 150°C, respectively, and the MS scanned at 70 eV between *m/z* 40 and 350.

Peaks were identified by spectral deconvolution with a target/non-target approach using Ion Analytics (Gerstel) software, as described previously (Kowalsick et al., 2014; Robbat et al., 2017; Kfoury et al., 2018a), using a database of reference mass spectra of 634 compounds previously detected in tea leaf samples (Kfoury et al., 2018b; Scott et al., 2019). The retention index (RI) of each compound was calculated using a standard mix of C7–C30 n-alkanes (Sigma-Aldrich) and used to confirm peak identity.

Relative peak areas (RPA) were calculated using the peak area of the internal standard. The RPA of compounds found in the field blank were subtracted and any compound where RPA ≤ 0 was replaced by the value 100/IS, where IS is the peak area of the internal standard. Any compounds detected in 5 or fewer samples were excluded from further analyses. RPAs were then natural log-transformed to improve normality. We tested for multivariate outliers using the *HDoutliers* package (Wilkinson, 2018) and removed one outlier (*n* = 18 for GC-MS data).

#### Sample Preparation and Analysis of Non-volatile Metabolites

Leaf samples were pulverized in a ball mill (KLECO, Visalia, CA, United States) and extracted in triplicate by mixing 20 ± 2 mg of powdered leaf material with 1 ml of 80:15:5 acetonitrile:water:1M HCl (v/v/v) in 1.5 ml Eppendorf microcentrifuge tubes. The microcentrifuge tubes were vortexed for 1 min under maximum setting. After vortexing, we sonicated the microcentrifuge tubes for 30 min at 20°C. The microcentrifuge tubes were then centrifuged for a minute at 1,300 g. The supernatant was decanted, and syringe filtered (0.45 μm PTFE membrane). The extracts were then stored at −20°C. Extracts were thawed and diluted 1:24 (40 μl extract and 960 μl extraction solvent) for measuring total phenolics and 1:9 for analysis by liquid chromatography-mass spectrometry (LC-MS).

The Folin-Ciocalteau assay was used to measure the total phenolic content of the tea extract as mg/g gallic acid equivalent (Unachukwu et al., 2010). Five gallic acid standard solutions (0.5, 0.25, 0.0625, and 0.03125 mg/ml) were prepared in 80:15:5 acetonitrile:water:1M HCl using serial dilution. The Folin-Ciocalteau assay was carried out in triplicate in a 96 well microtiter plate following Appel et al. (2001). All samples had coefficients of variation less than 20%. Triplicate results were averaged before analysis.

Analysis of specific non-volatiles was done using well established techniques (Scott et al., 2019). These non-volatiles included 8 catechins, 3 methylxanthine alkaloids, and 1 amino acid. Extracts for LC-MS analysis were spiked with 20 μg/ml paraxanthine (Sigma-Aldrich, ∼98%) as an internal standard. Non-volatile separations and quantitation were performed on a 1260 Infinity II HPLC (Agilent Technologies, Santa Clara, CA, United States) consisting of a quaternary pump, a chilled autosampler (4°C), a temperature-controlled column compartment, and a diode array detector (DAD) coupled with a 6120 quadrupole MS and electrospray ionization (ESI) source. 10 μl of sample was injected onto a superficially porous C18 column (150 mm × 3.0 mm i.d. × 2.7 mm d_p_, Agilent Technologies), with temperature set to 32°C. The mobile phase consisted of 0.1% formic acid in water (v/v) (A), and methanol (B). Separation was performed using a gradient elution program as follows: 6–35.7% B (0–22 min), 35.7–100% B (22–23 min), and held at 100% B for 5 min, with a re-equilibration time of 7 min, at a flow rate of 0.5 ml/min. The ESI used a drying gas temperature of 350°C, a flow rate of 12.0 l/min, a nebulizer pressure of 55 psig, and a capillary voltage of 3 kV. Mass spectra were acquired in positive ionization mode from *m/z* 100–500 at a rate of 0.943 spectra/sec, with fragmentation voltage of 130 V.

For calibration standards, we purchased a green tea standard (Sigma-Aldrich) containing 100 μg/ml each of (+)-catechin, (–)-catechin-3-gallate, (–)-epicatechin, (–)-epicatechin-3-gallate, (–)-epigallocatechin-3-gallate, (–)-gallocatechin, (–)-gallocatechin-3-gallate, and caffeine dissolved in 80:15:5 acetonitrile:water:1M HCL. In addition, L-theanine, theobromine, theophylline, and epigallocatechin were purchased in powdered form and dissolved in the same solvent as the green tea standard. Calibration samples were spiked with 10 μg/ml of paraxanthine internal standard (a compound not found in tea) and injected under conditions identical to the tea samples. Correlation coefficients for calibration curves ranged from 0.982 to 0.999. Ion Analytics software was used to analyze data files. Target compounds were quantified based on the [M + H]^+^ ion intensity, with compound identities confirmed using two qualifier fragment ions present at intensity ratios consistent (±20% relative intensity) with those observed in the standards, utilizing three ions total for detection. Triplicate measurements were averaged before statistical analysis.

### Statistics

All statistical analyses were conducted in R version 3.5.3 (R Core Team., 2018). To test for an effect of leafhopper herbivory we used two proxies of herbivore pressure: the density of leafhoppers at the end of the feeding period (insects/young leaf) and visible leaf damage (described in image analysis, above). Leaf damage was averaged across all leaves for non-volatile analysis since leaves were pooled for sampling. For volatile analysis, we present results from both mean leaf damage as well as damage to the focal DCSE leaf. Percent leaf damage was natural log-transformed for analysis to improve normality.

The effects of herbivory on total polyphenols were analyzed with linear models, but because of collinearity of metabolites, multivariate methods were used for LC-MS and GC-MS data. Redundancy analysis (RDA) was conducted on auto-scaled data (RPA for GC-MS and concentration for LC-MS) with either leafhopper density or damage as a continuous predictor variable using the *vegan* and *RVAideMemoire* packages (Hervé et al., 2018; Oksanen et al., 2018) as described in Hervé et al. (2018) (GC-MS *n* = 18, LC-MS *n* = 19). Metabolites with significant correlations with the first (only) RDA axis were considered biomarkers. For each biomarker, and total polyphenols, we fit univariate models with either natural log-transformed RPA (GC-MS) or concentration (LC-MS and total polyphenols) as the response variable and herbivory proxy as the predictor variable. Linear and intercept-only (null) models were fit with the *lm* function and step and hinge functions were fit with the *chngpt* package (Fong et al., 2017). For each compound, the best fit model was selected using a model competition approach with AIC (Akaike’s Information Criterion). Step function models were only considered significant when the 95% confidence interval for the change point did not overlap the minimum or maximum value for the predictor and when the change in intercepts was significantly different from zero.

## Results

### Herbivory

**FIGURE 1:**
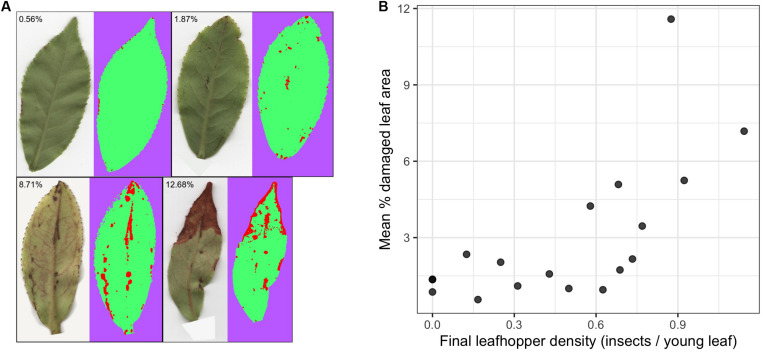
The relationship between leafhopper density and visible leaf damage. **(A)** Representative leaf images highlighting the range and types of damage with results from WEKA segmentation where red, green, and purple represent pixels that have been classified as damaged, undamaged, and background, respectively. **(B)** Scatter plots show the relationship between leafhopper density and mean percent leaf damage where each point represents a pot (*n* = 19). There is a threshold type relationship between leafhopper density and leaf damage.

Final leafhopper densities ranged from 0 to 1.14 insects/leaf (mean = 0.49, *SD* = 0.35) and mean percent damaged leaf area ranged from 0.57% to 11.58% (mean = 2.99, *SD* = 2.83) (Figure 1). There was a non-linear trend in the relationship between final leafhopper density and visible leaf damage (Figure 1B). Damage remained below 3% leaf area until about 0.6 insects/young leaf, when damage started to increase with increasing leafhopper density. Additionally, mean damage across all leaves and damage to the DCSE focal leaf were strongly correlated (Pearson’s *r* = 0.71).

### Volatiles

**FIGURE 2:**
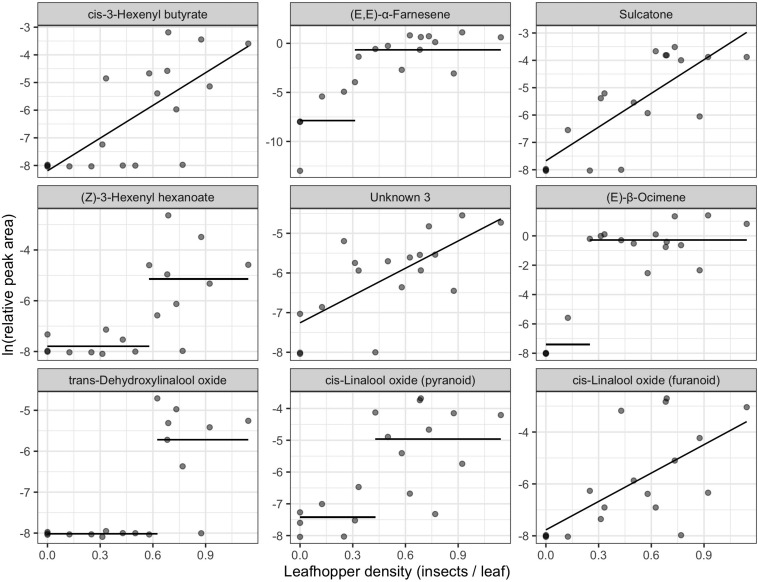
Biomarkers of leafhopper density identified from redundancy analysis of natural log-transformed relative peak areas of compounds detected in tea samples (*n* = 18). The x-axis represents the number of leafhoppers per young leaf at the end of the feeding period. Panels are in order of correlation to the RDA axis from strongest to weakest going left to right, top to bottom. Lines show fitted values of the winning univariate models. Only the top 9 biomarkers are plotted here. See Table 1 for a full list of biomarkers.

We found 155 volatile metabolites in total (Supplementary Table S1) with 76 compounds detected in all samples. Final leafhopper density explained 10.66% of the variation in volatile profile. There was a significant effect of final leafhopper density on the volatile profile [RDA, *F*_(1, 16)_ = 1.1, *p* = 0.002]. Step functions (a line with a slope of zero but a change in y-intercept at a threshold) best described the relationship between leafhopper density and natural log-transformed RPA for 12 of the biomarkers, linear models best described the relationship for 11 biomarkers, and an intercept-only model best explained the relationship for 12 of the biomarkers (Table 1). Linear and step function relationships for all biomarker compounds were positive (increasing RPA with increasing insect density) except for cyclopentanone. For compounds with linear relationships, slopes ranged from −2.22 to 3.94, and for compounds with step relationships the difference between intercepts ranged from 7.27 to 15.08. The compounds most strongly correlated with the RDA axis were *cis*-3-hexenyl butyrate, (*E*,*E*)-α-farnesene, sulcatone, (*Z*)-3-hexenyl hexenoate, unknown 3, (*E*)-β-ocimene, and three linalool oxides (Figure 2 and Table 1).

**TABLE 1:** Biomarkers of leafhopper density in order of the strength of correlation to the RDA constrained axis.

**Compound**	**Response type**	**Linear Slope**	**Change point**	**ΔIntercept**	**Aroma^a^**	**Biosynthesis**
*cis*-3-Hexenyl butyrate	Linear	3.941	–	–	Wine, green	FA derivative^b^
(*E,E*)-α-Farnesene	Step	–	0.312	7.21	Woody, sweet, green, floral	Sesquiterpene^c^
Sulcatone	Linear	4.11	–	–	Citrus, green, musty, cheesy	Irregular terpene^c^
(3Z)-Hexenyl hexanoate	Step	–	0.579	2.651	Green, fruity, fatty, tropical	FA derivative^b^
unknown 3	Linear	2.293	–	–	–	–
(*E*)-β-Ocimene	Step	–	0.25	7.119	Citrus, green, terpene	Monoterpene^c^
*trans*-Dehydroxylinalool oxide	Step	–	0.625	2.297	Herbal, green, terpene	Monoterpene^d^
*cis*-Linalool oxide (pyranoid)	Step	–	0.429	2.456	Citrus, green	Monoterpene^d^
*cis*-Linalool oxide (furanoid)	Linear	3.65	–	–	Earthy, floral, sweet, woody	Monoterpene^d^
(*E,E*)-Allo-ocimene	Step	–	0.25	3.065	Terpenic, sweet, fresh, floral	Monoterpene^c^
Indole	Step	–	0.682	1.601	Concentrated = fecal, animal dilute = sweet, floral	Anthranilate^b^
Diendiol I (2,6-Dimethylocta-3,7-diene-2,6-diol)	Linear	3.673	–	–	–	Monoterpene^e^
Benzyl alcohol	Linear	2.276	–	–	Fruity, floral, sweet	VPB^b^
β-Myrcene	Step	–	0.625	1.101	Balsamic, must, spice	Monoterpene^c^
*trans*-α-Bergamotene	Step	–	0.429	2.5	Woody, warm, tea	Sesquiterpene^c^
*cis*-3-Hexenyl isovalerate	Step	–	0.579	1.853	Fresh, green, apple, fruity, tropical	FA derivative^c^
Heptanoic acid	Null	–	–	–	Cheesy, sour, rancid	FA^f^
Hexanoic acid	Null	–	–	–	Cheesy, fatty	FA^f^
(*Z*)-β-Ocimene	Linear	3.321	–	–	Citrus, herbal, floral	Monoterpene^c^
(*E*)-2-Hexenyl acetate	Linear	1.676	–	–	Sweet, apple skin, banana peel	FA derivative^b^
1-Non-anol	Linear	1.716	–	–	Fatty, green, orange	FA derivative^b^
Decanal	Null	–	–	–	Citrus, sweet, waxy	–
Cyclopentenone	Null	–	–	–	–	FA derivative^g^
*trans*-Linalool oxide (furanoid)	Null	–	–	–	Earthy, floral, sweet, woody	Monoterpene^d^
γ-Non-alactone	Step	–	0.579	1.606	Coconut, creamy, waxy, sweet	FA derivative^h^
γ-Butyrolactone	Null	–	–	–	Caramel, fatty, sweet	FA derivative^h^
unknown 2	Null	–	–	–	–	–
p-Xylene	Linear	2.198	–	–	Sweet	–
1-Octen-3-ol	Null	–	–	–	Mushroomy, green, earthy	FA derivative^h^
Benzyl nitrile	Null	–	–	–	–	VPB^i^
Octanoic acid	Null	–	–	–	Cheesy, fatty, waxy	FA^f^
Butylated hydroxytoluene	Null	–	–	–	Phenolic, camphor	–
Linalool	Null	–	–	–	Flower, lavender	Monoterpene^c^
Cyclopentanone	Linear	-2.219	–	–	Minty	FA derivative^g^
Coumaran	Step	–	0.769	2.088	–	–

**For step function relationships, the change point, in units of leafhoppers per young leaf, and the difference in intercepts after and before the change point are reported. Biosynthetic pathways are reported when available from the literature (FA, fatty acid; VPB, volatile phenylpropanoid/benzenoid compounds derived from phenylalanine). ^*a*^The Good Scents Company, (2015), ^*b*^Dudareva et al. (2013), ^*c*^Knudsen et al. (2006), ^*d*^Ho et al. (2015), ^*e*^Zeng et al. (2019), ^*f*^Kroumova et al. (1994), ^*g*^Weber (2002), ^*h*^Schwab et al. (2008), ^*i*^Noge and Tamogami (2013).**

**FIGURE 3:**
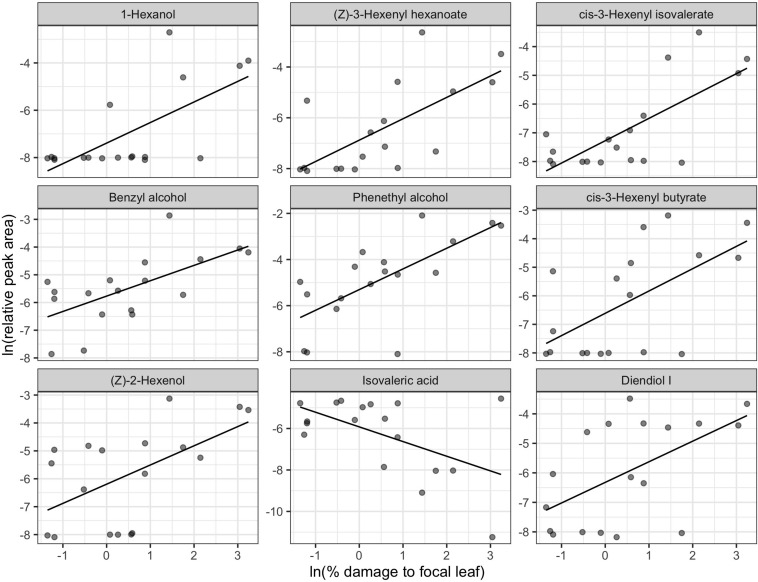
Biomarkers of leaf damage identified from redundancy analysis of natural log-transformed relative peak areas of compounds detected in tea samples (*n* = 18). The x-axis represents the natural log-transformed percentage of pixels classified as damaged on the leaf used for volatile sampling by DCSE. Panels are in order of correlation to the RDA axis from strongest to weakest going left to right, top to bottom. Lines show fitted values of the winning univariate models. Only the top 9 biomarkers are plotted here. The full chemical name for diendiol I is 2,6-Dimethylocta-3,7-diene-2,6-diol. See Table 2 for a full list of biomarkers.

We did not detect any significant relationship between natural log-transformed mean percent leaf damage and volatile profiles [RDA, *F*_(1, 16)_ = 1.132, *p* = 0.259]. However, the effect of focal leaf damage on volatile profile was marginally significant [RDA, *F*_(1, 16)_ = 1.329, *p* = 0.076] and explained 7.67% of the variation in volatiles. Linear relationships best described the shape of the relationship for 15 biomarkers, and intercept-only models best described the relationship for 7 biomarkers (Table 2). Most biomarkers had a positive relationship with focal leaf damage. Only isovaleric acid, *cis*-methyl dihydrojasmonate, and 1,2,4-trimethylbenzene showed a negative relationship with increasing focal leaf damage. The compounds most strongly correlated with the RDA axis were 1-hexanol; (*Z*)-3-hexenyl hexanoate; *cis*-3-hexenyl isovalerate; benzyl alcohol; phenylethyl alcohol; *cis*-3-hexenyl butyrate; (*Z*)-2-hexenol; isovaleric acid; and diendiol I (Figure 3).

**TABLE 2:** Biomarkers of visible leafhopper damage in order of the strength of correlation to the RDA constrained axis.

**Compound**	**Response type**	**Linear slope**	**Aroma**^a^	**Biosynthesis**
1-Hexanol	Linear	0.869	Resin, flower, green	FA derivative^b^
(*Z*)-3-Hexenyl hexanoate	Linear	0.842	Green, fruity, fatty, tropical	FA derivative^b^
*cis*-3-Hexenyl isovalerate	Linear	0.778	Green, apple, tropical, pineapple	FA derivative^b^
Benzyl alcohol	Linear	0.554	Fruity, floral, sweet	VPB^b^
Phenethyl alcohol	Linear	0.897	Honey, spice, rose, lilac	VPB^b^
*cis*-3-Hexenyl butyrate	Linear	0.782	wine, green	FA derivative^b^
(*Z*)-2-Hexenol	Linear	0.688	Leaf, green, wine, fruit	FA derivative^b^
Isovaleric acid	Step	-0.708	Sour, cheesy, rancid	FA^c^
Diendiol I	Linear	0.698	–	Monoterpene^d^
*cis*-Linalool oxide (pyranoid)	Linear	0.553	Citrus, green	Monoterpene^e^
(3-hydroxy-2,4,4-trimethylpentyl) 2-methylpropanoate	Null	–	–	–
Benzothiazole	Null	–	Rubbery, sulfury, vegetal, gasoline	–
γ-Non-alactone	Null	–	Coconut, creamy, waxy, sweet	FA derivative^f^
Indole	Null	–	Concentrated = fecal, animal dilute = sweet, floral	Anthranilate^b^
Coumaran	Linear	0.386	–	VPB^g^
*cis*-Linalool oxide (furanoid)	Null	–	Earthy, floral, sweet, woody	Monoterpene^e^
*trans*-Linalool oxide (furanoid)	Null	–	Earthy, floral, sweet, woody	Monoterpene^e^
1,2,4-Trimethylbenzene	Linear	-0.169	Plastic	VPB^g^
o-Hydroxybiphenyl	Null	–	–	–
Methyl salicylate	Linear	0.292	Wintergreen	VPB^g^
*cis*-Methyl dihydrojasmonate	Linear	-0.461	Jasmine, floral, green	FA derivative^g^
Benzyl nitrile	Null	–	–	VPB^h^

**Biosynthetic pathways are reported when available from the literature (FA, fatty acid; VPB, volatile phenylpropanoid/benzenoid compounds derived from phenylalanine). ^*a*^The Good Scents Company, (2015), ^*b*^Dudareva et al. (2013), ^*c*^Kroumova et al. (1994), ^*d*^Zeng et al. (2019), ^*e*^Ho et al. (2015), ^*f*^Schwab et al. (2008), ^*g*^Margl et al. (2005), ^*h*^Noge and Tamogami (2013).**

### Non-volatiles

**FIGURE 4:**
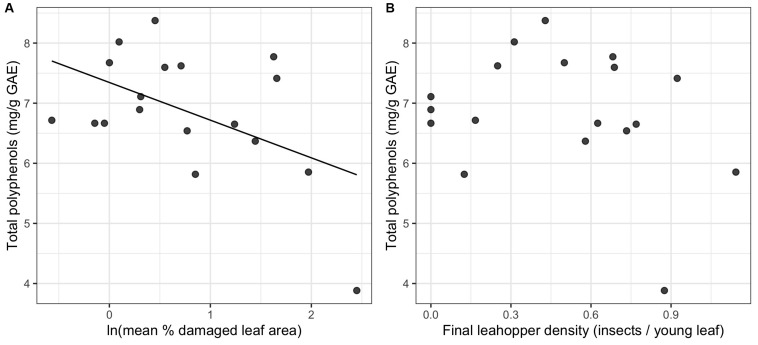
Relationship between total polyphenols and two proxies of herbivore damage: natural log-transformed mean% damaged leaf area **(A)** and leafhopper density **(B)** (*n* = 19). Only the relationship between leaf damage and total polyphenols in 2017 was statistically significant.

We did not detect a significant relationship between leafhopper density and total polyphenols (Figure 4B). However, for mean percent leaf damage, there was a significant negative linear relationship (ΔAIC > 2 for other model comparisons and *p* = 0.035) (Figure 4A).

**FIGURE 5:**
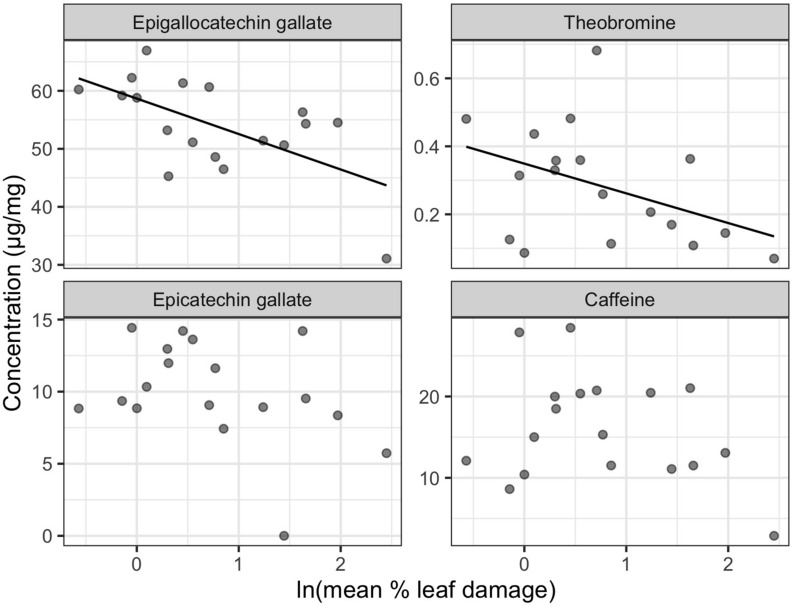
Non-volatile biomarkers for leafhopper damage. Only the LC-MS compounds with significant correlations to the RDA constrained axis are plotted (*n* = 19). Only epigallocatechin gallate and theobromine had a significant univariate linear relationship with natural log-transformed mean percent leaf damage.

Final leafhopper density explained only 5.44% of the total variation in LC-MS compound catechins, methylxanthines, and L-theanine) concentrations and did not have a significant effect [RDA, *F*_(1, 16)_ = 0.922, *p* = 0.449]. Although not statistically significant, mean percent damaged leaf area explained 9.58% of the total variation in LC-MS compound concentrations [RDA, *F*_(1, 16)_ = 1.696, *p* = 0.124]. Most compounds had negative loadings along the one significant RDA axis, which corresponds to decreasing concentrations with increasing leaf damage. Epigallocatechin gallate (EGCG), theobromine, epicatechin gallate, and caffeine were identified as biomarkers. EGCG and theobromine had significant negative linear relationships with leaf damage (Figure 5).

## Discussion

Few studies of induced secondary metabolites subject plants to a range of herbivore pressure despite evidence that different levels of herbivory produce different responses in plants. Here we investigated the effects of two quantitative proxies of *E. onukii* herbivory on tea plants: insect density and visible leaf damage. Interestingly, leafhopper density was a better predictor of plant chemical responses than was leaf damage, indicating that tea plants respond to leafhoppers even before their feeding damage is visibly detectible. The relationship between the two proxies, density and leaf damage, is not linear, and there are different metabolites associated with each of these proxies. In addition, we found a threshold effect for some, but not all, metabolites in response to leafhopper density.

There were non-linear relationships between our two proxies for herbivory (density and damage) as well as in the induced responses of volatiles to leafhopper density. The underlying mechanism explaining the variation in induced responses to leafhopper density may be related to induction of plant hormones. Liao et al. (2019) exposed tea plants to feeding by *E. onukii* for either 48 or 96 h and found that low herbivore pressure (48 h) induced jasmonic acid (JA) and salicylic acid (SA) but not abscisic acid (ABA), while high herbivore pressure induced JA and SA to a similar degree but additionally strongly induced ABA. The variation in these hormonal responses could not be explained by intensity of mechanical wounding alone. Non-linear responses of plant hormones in response to increasing herbivore pressure may be underlying the mix of dose-dependent and threshold responses we observed in our experiment. Future studies looking to explain the relationship between herbivore pressure and induced metabolites should consider measuring hormonal responses of plants to a continuous range of herbivore pressures.

### Estimating Herbivore Damage

Overall, our results suggest that increases in visible damage are not evident at low densities. We observed a non-linear relationship between our two proxies for herbivory, with a threshold of about 0.6 leafhoppers per young leaf before seeing an increase in visible damage. *Empoasca* species are known to be plastic in their feeding mode, switching between “burning,” which primarily targets leaf veins, and “stippling” which targets mesophyll (Backus et al., 2005; Jin et al., 2012). The non-linear relationship between insect density and visible damage could be a result of a density-dependent switch in feeding mode. A density-dependent switch in the response of the tea plant is also possible. For example, at low levels of herbivory there may be lower expression of PPO and therefore less browning of leafhopper feeding sites resulting in less visible feeding damage.

### Herbivory and Volatile Chemistry

Leafhopper density and visible damage are associated with different chemical profiles. The top biomarkers of leafhopper density were largely terpenes, with some contribution by fatty acid derivatives, such as green leaf volatiles, and some volatile phenylpropanoids/benzenoids (VPBs) (Table 1). Many of the volatile biomarkers of leafhopper density were compounds detected in previous studies of leafhopper feeding on tea (Gohain et al., 2012; Cai et al., 2014; Zeng et al., 2019). The shape of the relationship between leafhopper density and compound concentration (RPA) varied among compounds with induction being linearly density-dependent for some compounds or being induced only after a minimum threshold in herbivory for others. When step-functions were significant for biomarkers of leafhopper density, the threshold of induction was most often between 0.5 and 0.7 leafhoppers per leaf (Table 1), corresponding to the threshold at which we see mean leaf damage rise above 3% (Figure 1). These threshold-induced volatiles may be associated with whatever change is happening to produce an increase in visible damage, whether it is a change in feeding mode or a change in plant metabolic response. We note that the exact shape of the relationship between RPA and herbivory for any given compound depends on data transformation and may be partly influenced by the detection limits of the instrumentation. Regardless, the shape of the response of induced volatiles to leafhopper density varies among the entire suite of compounds analyzed, and this results in a change in volatile profile with increasing herbivore pressure.

Several of the compounds most strongly correlated with leafhopper density have been previously reported as being induced by insect feeding and serve as attractants for parasitoids or predators. Du et al. (1998) reported induction of (*E*)-β-ocimene, sulcatone (6-methyl-5-hepten-2-one), and linalool by pea aphids (*Acrothosiphon pisum*) feeding on broad bean (*Vicia faba*). Among the compounds they detected, sulcatone was most attractive to the parasitoid *Aphidius ervi*. They also reported that the concentration of (*E*)-β-ocimene was constant over 4 days of aphid feeding, while sulcatone, linalool, and other compounds increased in concentration with increased aphid feeding time. In our study, we see a similar pattern where (*E*)-β-ocimene is induced in a density-independent manner after a threshold of 0.25 leafhoppers per leaf, while sulcatone has a density-dependent relationship with leafhopper density (Table 1 and Figure 2). Interestingly, sulcatone is an oxidation product of (*E*,*E*)-α-farnesene in apples (Lurie and Watkins, 2012), so it is fascinating that they were not both linearly correlated with damage. This may indicate that induction of (*E*,*E*)-α-farnesene is less dependent on leafhopper density than it’s oxidation.

In contrast, our analysis of leaf damage revealed a different set of volatile biomarkers: largely fatty acid derivatives and VPBs, with a smaller contribution from terpenes (Table 2). Many of the volatile biomarkers related to visible leaf damage are commonly produced by plants as a result of mechanical damage to leaves by activation of the lipoxegenase (LOX) pathway including 1-hexanol, (*Z*)-3-hexenyl hexenoate, *cis*-3-hexenyl isovalerate, and (*Z*)-2-hexenol (Ho et al., 2015). It is likely that visible leaf damage is more closely associated to disruption of cell membranes compared to leafhopper density, which would explain why volatiles derived from free cell membrane lipids are correlated more strongly with this proxy for herbivory. In addition, the plant hormone methyl salicylate increases with increasing leaf damage in our study, consistent with the findings of Liao et al. (2019).

A biomarker of both leafhopper density and leaf damage, diendiol I, has been previously described as a compound induced in tea plants uniquely by *E. onukii* feeding (Zeng et al., 2019) and is a precursor to hotrienol, a compound formed during tea processing which is important for Eastern Beauty oolong flavor (Kawakami et al., 1995; Cho et al., 2007). Diendiol I increased linearly with increasing leafhopper density (and natural log-transformed leaf damage), although other volatile compounds that may impart off flavors also increased with leafhopper density. For example, sulcatone and *cis*-3-hexenyl butyrate increase at a steeper slope than diendiol I and impart green, musty, and vegetative odors (The Good Scents Company,, 2015).

Although volatile profile undergoes a dramatic change during tea processing, these results from live plants in the field have implications for finished tea quality (Cho et al., 2007). In addition to inducing important chemical precursors (i.e., diendiol I), leafhopper herbivory may potentially prime tea leaves to respond differently to the stresses experienced during the oxidation phase of oolong tea processing (Zeng et al., 2019). In order to fully understand how environmental factors such as insect herbivory affect the quality of tea, more studies are needed that track tea metabolites in mature tea plants in the field, through processing, and in finished tea.

Because metabolites differ in their response to increasing leafhopper herbivory, and different proxies for herbivory are associated with different metabolites, volatile profiles change with increasing leafhopper herbivory in complex ways. This has implications for leafhoppers and their natural enemies which both use plant volatiles to locate suitable food (Gao et al., 2004; Xin et al., 2017). For example, future studies could examine how predator or parasitoid behavior changes with exposure to volatile profiles produced by tea plants above or below the threshold of leafhopper density we’ve observed.

### Herbivory and Non-volatile Chemistry

In contrast to volatiles, variation in non-volatiles (including catechins and total polyphenols) was explained by visible damage rather than by leafhopper density. We detected a significant reduction in epigallocatechin gallate (EGCG) and total polyphenols with increasing leaf damage, but no relationship with leafhopper density, which is consistent with the prediction that visible damage is due to increased polyphenol oxidase (PPO) activity producing brown theaflavins and thearubigins from catechin precursors (Tounekti et al., 2013). Liao et al. (2019) observed an increase in PPO activity and an increase in theaflavins (the oxidation products of catechins) with *E. onukii* damage on tea, although they saw no decrease in catechin concentrations. In contrast to our results, Li et al. (2018) found a quadratic relationship between total polyphenol content and *E. onukii* density with the highest polyphenol concentration at an intermediate level of herbivory corresponding to 2 insects per leaf. Theobromine, a caffeine precursor (Xia et al., 2017), also decreased in concentration with leafhopper damage in our study. Previous research showed an increase in caffeine synthase gene expression after leafhopper feeding, but no increase in caffeine concentration was detected (Yang et al., 2011; Liao et al., 2019). Theobromine concentrations were not measured in either of these studies, although it is possible that theobromine reduction is associated with increased caffeine synthase expression.

Decreases in polyphenols and EGCG may not be desirable for tea farmers, as these compounds are beneficial for health (Pon Velayutham, 2008). However, polyphenols can also contribute bitterness and astringency to tea, which may decrease quality from a flavor standpoint (Scott and Orians, 2018). In addition, the non-volatile and volatile metabolite profiles of tea changes with tea processing (Chengying et al., 2018; Jiang et al., 2019). So while our study shows that leafhopper herbivory has implications for finished tea quality, the exact effects of herbivore density and damage on taste, aroma, and health beneficial compounds have yet to be elucidated.

## Summary

We show that leafhoppers affect tea plant metabolites in complex ways as the intensity of herbivory increases. Although the effects of climate change on *E. onukii* are currently unknown, their population densities are likely to increase, resulting in changes in tea metabolites. As a highly multivoltine species, increases in the length of the growing season may allow for additional generations and an increase in numbers (Berggren et al., 2009; Reineke and Hauck, 2012). This, in turn, could have implications for the practice of taking advantage of natural levels of leafhopper herbivory to improve tea quality.

As importantly, the two methods used for quantifying herbivore pressure are associated with different lists of biomarkers, and thus suggest that plant responses to damage are complex. Thus, gathering data on multiple proxies for herbivore pressure can be key to elucidating plant responses to herbivory. For example, Lin et al. (1990) found that behavioral responses of beetles to plants damaged by conspecifics depended on the number of wounded cells in contact with healthy cells and not on the area of plant tissue removed by herbivory. Similarly, the density of herbivores, the area of damaged tissue, and feeding time (not manipulated in this study, but used as a dependent variable in many others) may be non-equivalent measures of herbivore pressure on plants. When possible, multiple proxies for herbivory can be measured and any disagreement in the results using these proxies should be viewed as an opportunity to better understand the underlying biological and methodological mechanisms that lead to contrasting results.

Finally, our results show that treating herbivory as continuous rather than categorical (e.g., control vs. damaged), has the potential to reveal important non-linear responses to herbivory. These non-linear responses may be ecologically relevant if they result in a different behavior of conspecifics or predators after a certain threshold of herbivory, and they may be economically relevant when induced secondary metabolites contribute to crop quality. Future studies of induced responses to herbivory should, at minimum, include more than one level of herbivory and ideally account for natural variation in herbivore feeding by measuring actual herbivory quantitatively.

## Data Availability Statement

The datasets analyzed for this study can be found in the following Zenodo archive: http://doi.org/10.5281/zenodo.3614045.

## Author Contributions

ES, W-YH, J-PW, XL, and CO contributed to the conception and design of the study. SA, TG, SC, and JS provided input on study design and field site selection. W-YH and XL secured field sites and tea plants for the study. ES, J-PW, M-MG, and XL carried out the field experiments. NK, JM, AR, and AA performed the chemical analyses. ES and AA carried out the image analysis. ES wrote the manuscript with contributions from SA, NK, CO, XL, SC, TG, and JS.

## Conflict of Interest

The authors declare that the research was conducted in the absence of any commercial or financial relationships that could be construed as a potential conflict of interest.
