# Supplementary figures and images for: Changes in Tea Plant Secondary Metabolite Profiles as a Function of Leafhopper Density and Damage
Source: Front Plant Sci. 2020 May 29;11:636. doi: 10.3389/fpls.2020.00636 (PMC7272924; doi:10.3389/fpls.2020.00636)

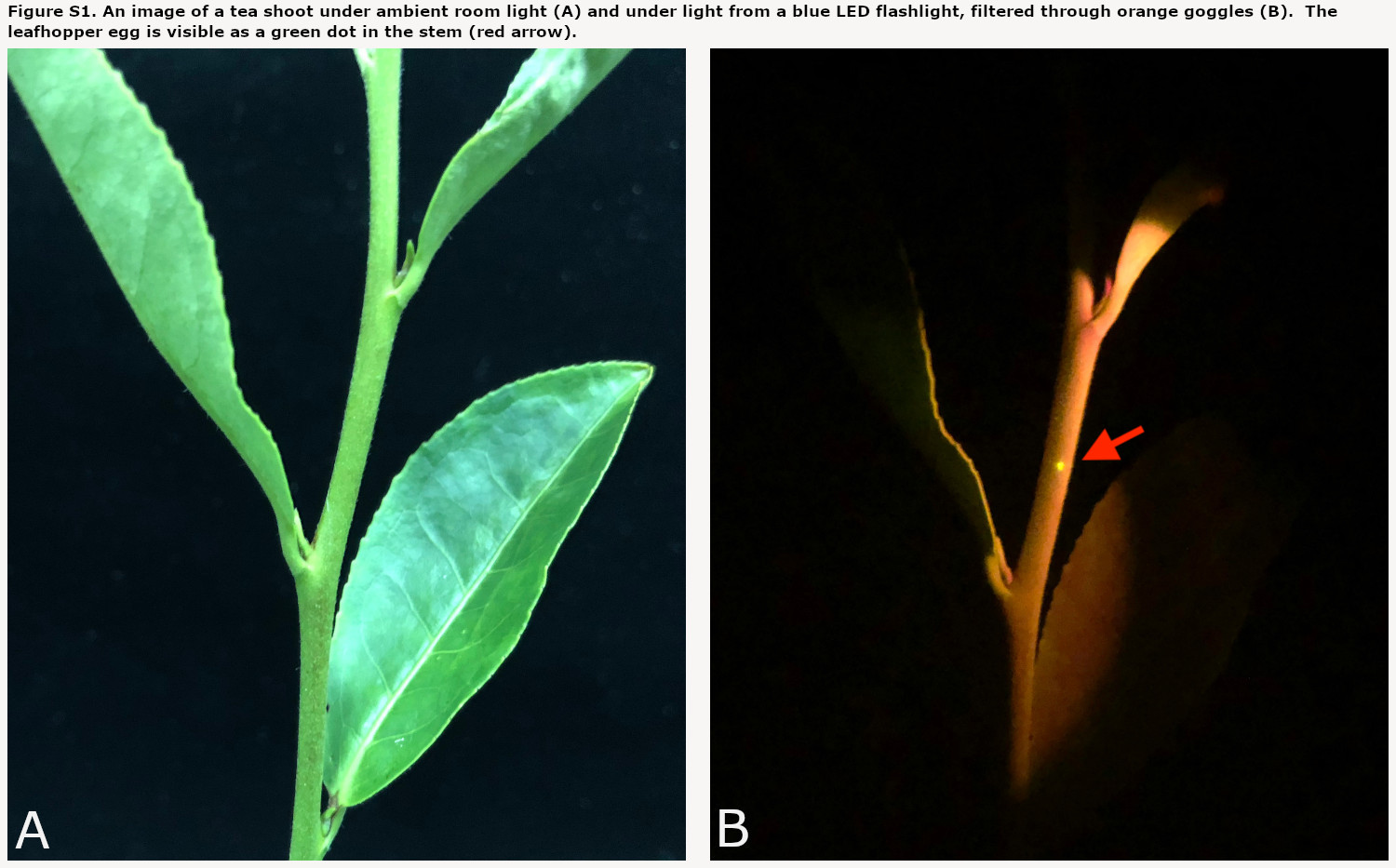

Supplement: Supplementary file 1 [file Image_1.JPEG]
